# fSCIG 10% in pediatric primary immunodeficiency diseases: a European post-authorization safety study

**DOI:** 10.1186/s13223-024-00904-9

**Published:** 2024-09-17

**Authors:** Peter Čižnár, Marion Roderick, Helen Schneiderova, Miloš Jeseňák, Gergely Kriván, Nicholas Brodszki, Stephen Jolles, Charles Atisso, Katharina Fielhauer, Shumyla Saeed-Khawaja, Barbara McCoy, Leman Yel

**Affiliations:** 1 Department of Paediatrics, Faculty of Medicine, Comenius University Bratislava, National Institute of Children’s Diseases, Bratislava, Slovakia; 2 Department of Paediatric Immunology, Bristol Royal Hospital for Children, Bristol, UK; 3 Department of Pediatrics, Faculty of Medicine, Masaryk University, Brno, Czech Republic; 4 Centre for Primary Immunodeficiencies, Department of Pediatrics, Jessenius Faculty of Medicine, Comenius University in Bratislava, University Hospital Martin, Martin, Slovakia; 5 Department of Pediatric Hematology & Stem Cell Transplantation, Central Hospital of Southern Pest, National Institute of Hematology and Infectious Diseases, Budapest, Hungary; 6 Department of Pediatric Oncology, Hematology and Immunology, Skåne University Hospital, Lund, Sweden; 7 Immunodeficiency Centre for Wales, University Hospital of Wales, Cardiff, UK; 8 Takeda Development Center Americas, Inc., Cambridge, MA USA; 9 Baxalta Innovations GmbH, a Takeda Company, Vienna, Austria; 10 University of California Irvine, Irvine, CA USA

**Keywords:** Hyaluronidase, Immunoglobulins, Inborn errors of immunity (IEI), Primary immunodeficiency diseases, Patient safety, Pediatrics, Subcutaneous

## Abstract

**Background:**

The safety, tolerability, and immunogenicity of hyaluronidase-facilitated subcutaneous immunoglobulin (fSCIG) 10% (dual-vial unit of human immunoglobulin 10% and recombinant human hyaluronidase [rHuPH20]) were assessed in children with primary immunodeficiency diseases (PIDs).

**Methods:**

This phase 4, post-authorization, prospective, interventional, multicenter study (NCT03116347) conducted in the European Economic Area, enrolled patients aged 2 to < 18 years with a documented PID diagnosis who had received immunoglobulin therapy for ≥ 3 months before enrollment. New fSCIG 10% starters underwent fSCIG 10% dose ramp-up for ≤ 6 weeks (epoch 1) before receiving fSCIG 10% for ≤ 3 years (epoch 2); patients pretreated with fSCIG 10% entered epoch 2 directly. The primary outcome was the number and rate (per infusion) of all noninfectious treatment-related serious and severe adverse events (AEs).

**Results:**

In total, 42 patients were enrolled and dosed (median [range] age: 11.5 [3–17] years; 81% male; 23 new starters; 19 pretreated). Overall, 49 related noninfectious, treatment-emergent AEs (TEAEs) were reported in 15 patients; most were mild in severity (87.8%). No treatment-related serious TEAEs were reported. Two TEAEs (infusion site pain and emotional distress) were reported as severe and treatment-related in a single new fSCIG 10% starter. The rate of local TEAEs was lower in pretreated patients (0.1 event/patient-year) versus new starters (1.3 events/patient-year). No patients tested positive for binding anti-rHuPH20 antibodies (titer of ≥ 1:160).

**Conclusions:**

No safety signals were identified, and the incidence of local AEs declined over the duration of fSCIG 10% treatment. This study supports fSCIG 10% long-term safety in children with PIDs.

**Trial registration number (ClinicalTrials.gov):**

NCT03116347.

**Supplementary Information:**

The online version contains supplementary material available at 10.1186/s13223-024-00904-9.

## Introduction

Patients with primary immunodeficiency diseases (PIDs), recently referred to as inborn errors of immunity (IEI) [1], are susceptible to recurrent and opportunistic infections owing to defects in humoral and cell-mediated immunity [2, 3]. To avert or reduce infection severity, and prevent related morbidity and even mortality, patients with PIDs with associated antibody deficiencies require early diagnosis and often long-term or lifelong treatment with immunoglobulin replacement therapy (IgRT). IgRT remains the fundamental pillar of treatment for most patients with PIDs [4–6].

Hyaluronidase-facilitated subcutaneous immunoglobulin (fSCIG) 10% (HyQvia [Baxalta US, Inc., a Takeda company, Cambridge, MA, USA]) is a dual-vial unit of immunoglobulin G (IgG) 10% and recombinant human hyaluronidase (rHuPH20), approved in the European Union as IgRT for adults and pediatric patients (aged 0–18 years) with PIDs or secondary immunodeficiency diseases [7], and in the USA to treat PIDs in adults and children aged ≥ 2 years [8]. rHuPH20 depolymerizes hyaluronan in the extracellular matrix, transiently increasing subcutaneous tissue permeability to IgG and allowing for high-volume subcutaneous IgG administration within a relatively short time [9]. The opportunity to administer increased subcutaneous IgG volumes enables longer treatment intervals than conventional subcutaneous immunoglobulin (SCIG) treatments, and similar to intravenous immunoglobulin (IVIG).

Previous studies, including a pivotal phase 3 study (NCT00814320), have shown fSCIG 10% to be effective and bioequivalent to IVIG, with fewer systemic adverse events (AEs) and more stable trough IgG plasma levels in both adults and children with PIDs [10–12]. fSCIG 10% has also demonstrated a favorable tolerability profile in patients experiencing systemic AEs following IVIG [13]. In the pivotal analysis, fSCIG 10% infusions were well tolerated, and sustained serum IgG trough levels were observed, with patients experiencing low rates of infection and of local and systemic reactions [10]. Additionally, results from an analysis of a phase 3 US study (NCT03277313) provide further supporting evidence of the favorable long-term safety profile of fSCIG 10% in children with PIDs [14].

This post-authorization safety study investigated the long-term safety, tolerability, and immunogenicity of fSCIG 10% in pediatric patients (aged 2 to < 18 years) with PIDs in Europe. Efficacy, administration characteristics, and fSCIG 10% impact on health-related quality of life (HRQoL) and healthcare resource utilization (HCRU) were also assessed.

## Methods

### Study design

This phase 4, post-authorization, prospective, interventional, noncontrolled study (NCT03116347) was conducted at 16 centers in the European Economic Area. The first patient enrolled on May 30, 2017 and the last patient completed the study on January 15, 2021.

**Fig. 1:**
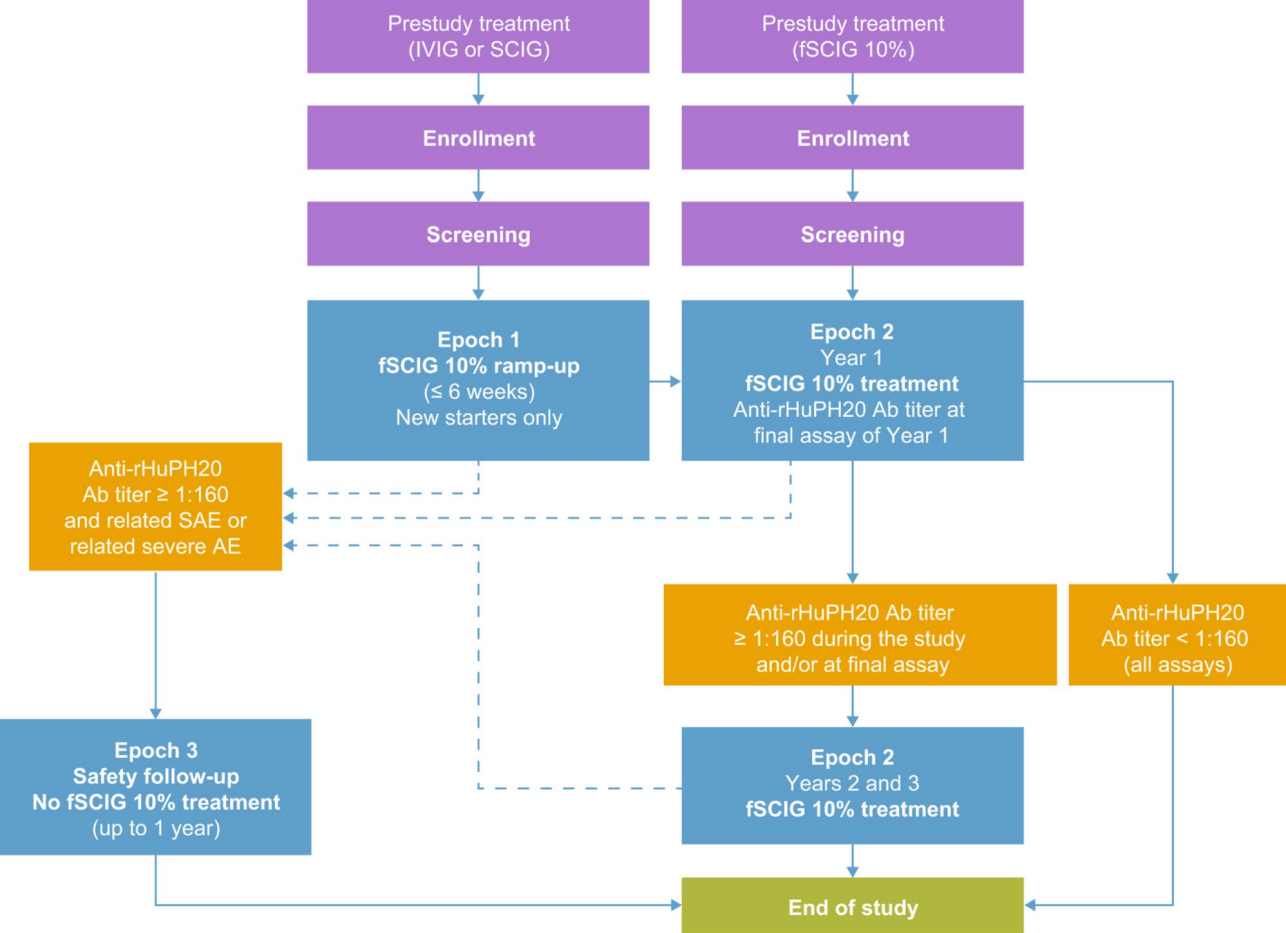
Study design. Epoch 1 (fSCIG 10% ramp-up): ≤ 6 weeks. Epoch 2 (fSCIG 10% treatment): 1–3 years. Epoch 3 (safety follow-up): up to 1 year. Ab, antibody; AE, adverse event; fSCIG, hyaluronidase-facilitated subcutaneous immunoglobulin; IVIG, intravenous immunoglobulin; rHuPH20, recombinant human hyaluronidase; SAE, serious adverse event; SCIG, subcutaneous immunoglobulin

The study comprised three epochs (Fig. 1). In epoch 1, patients previously receiving IVIG or SCIG were treated for the first time with fSCIG 10% at the study site, with a dose ramp-up period of ≤ 6 weeks (hereafter referred to as “new starter” patients). Patients already receiving fSCIG 10% before enrollment directly entered epoch 2 (hereafter referred to as “pretreated” patients). During epoch 2, for IVIG-pretreated patients, fSCIG 10% was administered every 3 or 4 weeks depending on the patient’s previous IVIG regimen; for SCIG-pretreated patients, fSCIG 10% was administered every 3 or 4 weeks at investigator and patient discretion.

In epochs 1 and 2, the fSCIG 10% dose was calculated in grams per kilogram (g/kg) based on latest patient weight. After 1 year in epoch 2, anti-rHuPH20 binding antibody assay results determined next steps for individual patients. Patients with anti-rHuPH20 binding antibody titers of < 1:160 (predefined threshold for a positive antibody test) underwent the study completion visit at the next possible occasion. Patients with anti-rHuPH20 binding antibody titers of ≥ 1:160 during the study and/or at the last measurement remained in epoch 2 for an additional 2 years of fSCIG 10% treatment and observation.

Patients with an anti-rHuPH20 binding antibody titer of ≥ 1:160 during epochs 1 or 2 who experienced either a treatment-related serious AE (SAE) or a severe AE underwent further safety follow-up for up to 1 year in epoch 3. During this time, instead of fSCIG 10%, patients received IVIG 10% (human normal immunoglobulin solution for infusion; Kiovig, Baxalta Innovations GmbH, a Takeda company, Vienna, Austria) or SCIG 20% (Cuvitru, Baxalta US, Inc., a Takeda company, Cambridge, MA, USA) at investigator and patient discretion. Patients in epoch 3 underwent regular anti-rHuPH20 binding antibody testing (approximately every 3 months) and underwent the study completion visit either upon resolution of the previous SAE or severe AE, or when anti-rHuPH20 binding antibody titers were < 1:2560.

Patients in epochs 1 or 2 experiencing treatment-related SAEs or severe AEs without an anti-rHuPH20 antibody titer of ≥ 1:160 were, at investigator or patient discretion, discontinued from the study, directly switched to epoch 3, or continued in epochs 1 or 2 (as applicable) with appropriate medical intervention.

### Patient eligibility

Eligible patients met the following criteria for enrollment: aged 2 to < 18 years with a documented PIDs diagnosis involving an antibody formation defect requiring IgRT; had received IgG for at least 3 months at a consistent dose before screening; and had a serum IgG trough level > 5 g/L at screening. Informed consent had to be provided by parents or caregivers. Patients were excluded if they had an active infection and were receiving antibiotic therapy for infection at screening. Full study exclusion criteria are presented in Supplementary methods, Additional file 1.

### Study outcomes

The primary outcome was the safety of fSCIG 10% determined by the number and rate per infusion and per patient-year of all noninfectious treatment-related SAEs and severe AEs. Secondary and tertiary outcome measures included additional safety outcomes (including number of infections and acute serious bacterial infections [ASBIs]), tolerability, immunogenicity, efficacy (assessed by serum IgG trough levels), treatment administration characteristics, HRQoL, and HCRU. A serum IgG trough level of 7 g/L was considered a putative therapeutic protective threshold against infection (further outcome measure details given in Supplementary methods, Additional file 1) [15].

### Statistical analysis

The number of patients deemed sufficient to collect safety data from this population was approximately 40. Two analysis populations were defined: the full analysis set (FAS; all patients providing informed consent and meeting enrollment eligibility) and the safety analysis set (SAS; all patients from the FAS receiving at least one dose of fSCIG 10% after enrollment). Safety analysis was based on the SAS. Primary outcome measures were descriptively analyzed.

### Ethics and study conduct

Before patient participation, the study protocol, final informed consent form, any promotional material/advertisements, and any other written information were reviewed and approved by the Ethics Committee and applicable regulatory authorities. This study was conducted in accordance with the International Council for Harmonisation Guideline for Good Clinical Practice E6 (ICH GCP R2, November 2016), Title 21 of the US Code of Federal Regulations, the European Union Directives 2001/20/EC and 2005/28/EC, the Declaration of Helsinki, and applicable national and local regulatory requirements. All patients, parents, or caregivers provided informed consent before inclusion in the study.

## Results

### Patient disposition and characteristics

**Fig. 2:**
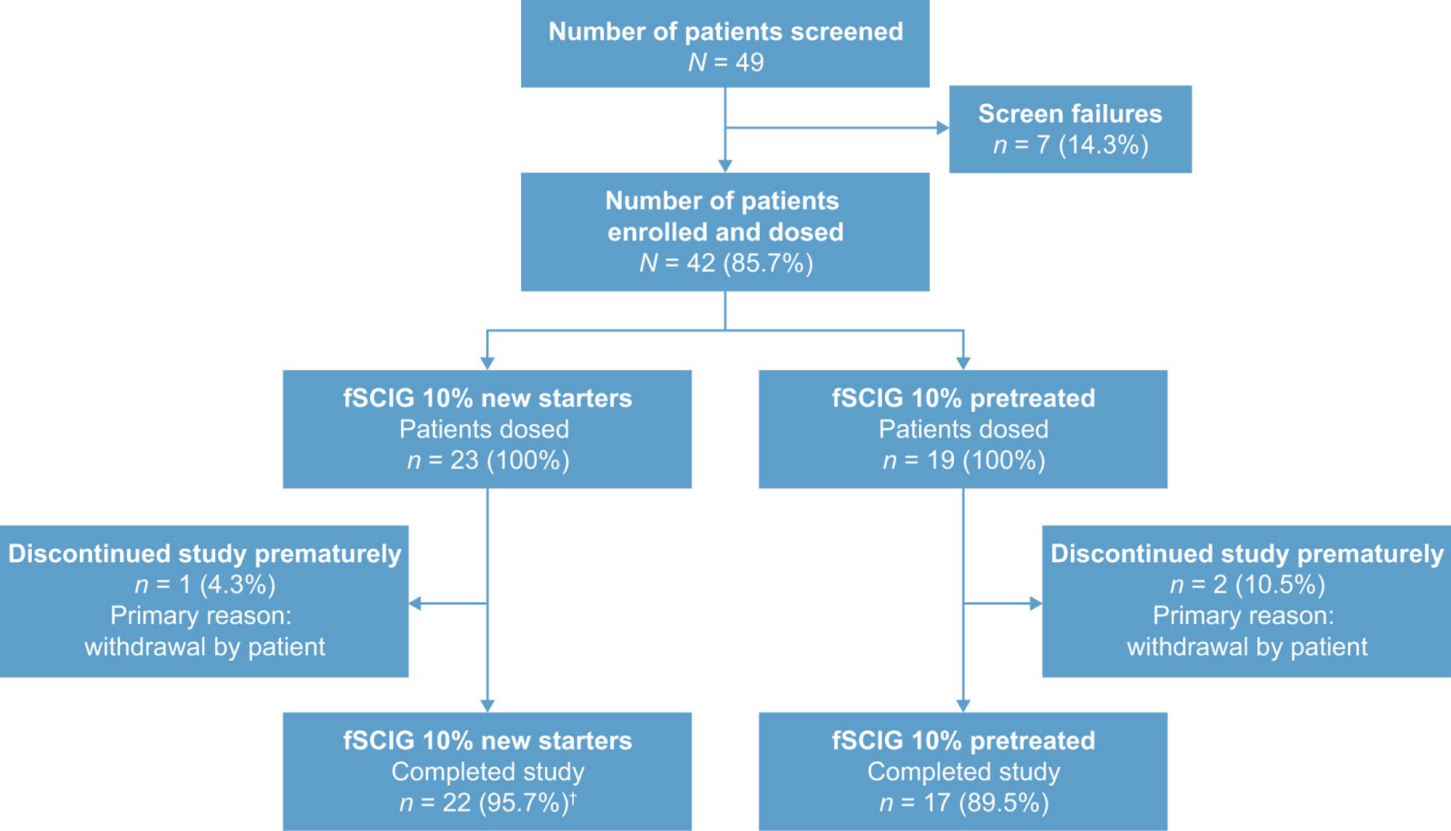
Patient disposition. ^†^22 of 23 new starters completed epoch 1, and continued and completed epoch 2. fSCIG, hyaluronidase-facilitated subcutaneous immunoglobulin

Overall, 49 patients were screened, of whom 42 were enrolled and dosed with study treatment; 23 new starters entered epoch 1, and 19 pretreated patients entered epoch 2 (Fig. 2). The overall mean (standard deviation [SD]) duration in the study was 15.5 (3.6) months, with a mean (SD) fSCIG 10% exposure of 14.4 (4.0) months. Baseline characteristics were broadly similar between new starters and pretreated patients (Table 1).

**Table 1:** Baseline demographics and disease characteristics

	fSCIG 10% new starters (*n =* 23)	fSCIG 10% pretreated (*n =* 19)	Overall (*N =* 42)
Age, years^†^			
Mean (SD)	10.3 (3.8)	11.7 (4.3)	11.0 (4.1)
Median (range)	11.0 (3–17)	12.0 (3–17)	11.5 (3–17)
Age group, years, *n* (%)			
2 to < 6 years	3 (13.0)	3 (15.8)	6 (14.3)
6 to < 12 years	11 (47.8)	4 (21.1)	15 (35.7)
12 to < 18 years	9 (39.1)	12 (63.2)	21 (50.0)
Male sex, *n* (%)	18 (78.3)	16 (84.2)	34 (81.0)
Ethnicity, *n* (%)			
Not Hispanic or Latino	23 (100)	19 (100)	42 (100)
Race, *n* (%)			
White	22 (95.7)^‡^	19 (100)	41 (97.6)^‡^
BMI, kg/m^2^			
Mean (SD)	19.0 (4.3)	20.4 (5.1)	19.7 (4.6)
Median (range)	18.3 (12.3–30.3)	18.8 (13.5–32.7)	18.6 (12.3–32.7)
PIDs diagnosis, *n* (%)			
Agammaglobulinemia – AR	1 (4.3)	1 (5.3)	2 (4.8)
Congenital agamma – XLA	8 (34.8)	8 (42.1)	16 (38.1)
Common variable immunodeficiency	11 (47.8)	7 (36.8)	18 (42.9)
Severe combined immune deficiency	0	1 (5.3)	1 (2.4)
Activated phosphokinase 3 delta receptor syndrome^§^	0	1 (5.3)	1 (2.4)
Hypogammaglobulinemia^§^	1 (4.3)	0	1 (2.4)
NEMO immune deficiency^§^	2 (8.7)	0	2 (4.8)
PI3K-delta syndrome^§^	0	1 (5.3)	1 (2.4)

^†^Age at baseline (last non-missing value before initial dose of fSCIG 10%)

^‡^Race for one patient could not be collected as per local regulations

^§^These diagnosis terms were provided by the reporting clinician when listing “Other” PIDs diagnoses

AR, autosomal recessive; BMI, body mass index; fSCIG, hyaluronidase-facilitated subcutaneous immunoglobulin; NEMO, nuclear factor kappa B essential modulator; PI3K, phosphoinositide 3-kinase; PIDs, primary immunodeficiency diseases; SD, standard deviation; XLA, X-linked agammaglobulinemia

### Primary outcome: noninfectious treatment-related serious or severe adverse events

No noninfectious or infectious treatment-related SAEs were reported across the entire study. Only two severe treatment-related treatment-emergent AEs (TEAEs) (infusion site pain, emotional distress) were reported in a new starter who discontinued epoch 2, with an overall rate of < 0.1 events per infusion, per patient, and per patient-year. Both events were noninfectious, and the patient had a history of self-harming and several ongoing medical conditions including developmental delay, anxiety, and attention deficit hyperactivity disorder. Following discontinuation in epoch 2, the patient entered epoch 3 and went on to complete the study (see Supplementary results, Additional file 1 for additional details).

### Other safety and tolerability outcomes

In total, 152 noninfectious TEAEs were reported in 29 patients (69.0%) (Table 2); 102 TEAEs were reported in 18 new starters (78.3%) and 50 in 11 pretreated patients (57.9%). Forty-one local noninfectious TEAEs, of which 36 (87.8%) were mild, were reported in 14 patients (33.3%), and 111 systemic TEAEs, of which 95 (85.6%) were mild, were reported in 25 patients (59.5%).

**Table 2:** Summary of noninfectious treatment-emergent adverse events

	fSCIG 10% new starters (*n =* 23)		fSCIG 10% pretreated (*n =* 19)		Total (*N =* 42)	
	Patients, *n* (%)	Events, *n*	Patients, *n* (%)	Events, *n*	Patients, *n* (%)	Events, *n*
AEs (all causalities)						
Any	18 (78.3)	102	11 (57.9)	50	29 (69.0)	152
Local	11 (47.8)	38	3 (15.8)	3	14 (33.3)	41
Systemic	15 (65.2)	64	10 (52.6)	47	25 (59.5)	111
Serious	0 (0.0)	0	3 (15.8)	4	3 (7.1)	4
**Treatment-related AEs**						
Any	13 (56.5)	52	6 (31.6)	11	19 (45.2)	63
Local	11 (47.8)	38	3 (15.8)	3	14 (33.3)	41
Systemic	6 (26.1)	14	4 (21.1)	8	10 (23.8)	22
Serious	0 (0.0)	0	0 (0.0)	0	0 (0.0)	0
Severe	1 (4.3)	2	0 (0.0)	0	1 (2.4)	2
**Most common AEs** ^†^						
Local^‡^						
Infusion site pain	7 (30.4)	15	1 (5.3)	1	8 (19.0)	16
Infusion site pruritus	5 (21.7)	7	0 (0.0)	0	5 (11.9)	7
Infusion site erythema	3 (13.0)	3	0 (0.0)	0	3 (7.1)	3
Systemic^§^						
Cough^II^	8 (34.8)	13	4 (21.1)	8	12 (28.6)	21
Pyrexia^II^	3 (13.0)	8	3 (15.8)	8	6 (14.3)	16
Vomiting	3 (13.0)	5	1 (5.3)	2	4 (9.5)	7
Epistaxis	2 (8.7)	2	1 (5.3)	5	3 (7.1)	7
Fatigue	3 (13.0)	3	1 (5.3)	2	4 (9.5)	5
Oropharyngeal pain	1 (4.3)	2	2 (10.5)	2	3 (7.1)	4
Rhinorrhea	2 (8.7)	2	1 (5.3)	1	3 (7.1)	3

^†^Reported in more than two patients overall

^‡^All were considered to be related to fSCIG 10% treatment

^§^Events considered related/temporally associated (occurring during or within 72 hours of infusion) with fSCIG 10% treatment were cough (2 events: 1 each in 1 new starter and 1 pretreated patient), vomiting (4 events: 2 each in 1 new starter and 1 pretreated patient), pyrexia (7 events: 5 events in 2 new starters and 2 events in 1 pretreated patient), fatigue (1 event in 1 new starter), rhinorrhea (1 event in 1 new starter)

^II^Events of cough and pyrexia coincided in two instances, but neither instance was associated with documented infection. In total, four events of pyrexia and two events of cough coincided with additional diagnosis terms captured as “Infections and Infestations”, according to MedDRA System Organ Class terms

AE, adverse event; fSCIG, hyaluronidase-facilitated subcutaneous immunoglobulin; MedDRA, Medical Dictionary for Regulatory Activities

Rates of noninfectious TEAEs per infusion, per patient, and per patient-year were 0.2, 3.6, and 2.9, respectively. Overall local TEAE rates per infusion, per patient, and per patient-year were < 0.1, 1.0, and 0.8, respectively, which were higher among new starters (< 0.1, 1.7, and 1.3, respectively) than pretreated patients (< 0.1, 0.2, and 0.1, respectively). The most frequently reported local AE was infusion site pain, predominantly in new starters (Table 2). Systemic TEAE rates were comparable between patient groups (new starters: 2.2 events per patient-year; pretreated patients: 2.0 events per patient-year). The most commonly reported individual systemic AEs were cough and pyrexia across both groups (Table 2).

In total, 49 noninfectious adverse reactions were reported in 15 patients (35.7%) with a rate per infusion of < 0.1, with higher numbers reported in new starters (42 events in 12 patients; 52.2%) than in pretreated patients (7 events in 3 patients; 15.8%).

A total of 63 treatment-related/temporally associated TEAEs were reported in 19 patients (45.2%), with a rate per infusion of < 0.1, and with a lower overall number of events in pretreated individuals (11 events in 6 patients; 31.6%) than in new starters (52 events in 13 patients; 56.5%).

Overall, 8 serious TEAEs (including infections) were reported in 7 patients (16.7%), consisting of one event each of acute sinusitis, dental caries, idiopathic orbital inflammation, inflammatory bowel disease, pharyngitis, pilonidal cyst, pneumonia, and pyrexia.

### Immunogenicity

No patients developed binding anti-rHuPH20 antibodies with a titer of ≥ 1:160; therefore, no individuals were assessed for neutralizing anti-rHuPH20 antibodies.

### Infections

Overall, 78 treatment-emergent infections were reported in 32 patients (76.2%), with 46 reported in 17 new starters (73.9%) and 32 reported in 15 pretreated patients (78.9%).

Rates of all infections per infusion and per patient-year were < 0.1 and 1.5, respectively, with rates similar across both fSCIG 10% new starters and pretreated patients. The most frequently reported infections (occurring in ≥ 10% of patients across groups) were rhinitis (14 events in 8 patients [19.0%]), gastroenteritis (7 events in 5 patients [11.9%]), and nasopharyngitis (6 events in 5 patients [11.9%]).

Only a single ASBI (bacterial pneumonia) was reported in the entire study (< 0.1 event per patient-year). This occurred in a new fSCIG 10% starter (male, aged 16 years at the time of event) who was hospitalized owing to pneumonia. This event was of moderate severity and assessed by the site investigator as unrelated to fSCIG 10%, with the patient’s underlying agammaglobulinemia proposed as an alternative etiology. The infection was treated using intravenous rehydration therapy and antibiotics (14-day course of oral ciprofloxacin 400 mg twice daily). The patient’s serum IgG trough level closest to the timing of the event was 8.12 g/L (48 days prior to onset of the ASBI).

### Serum IgG trough levels

**Fig. 3:**
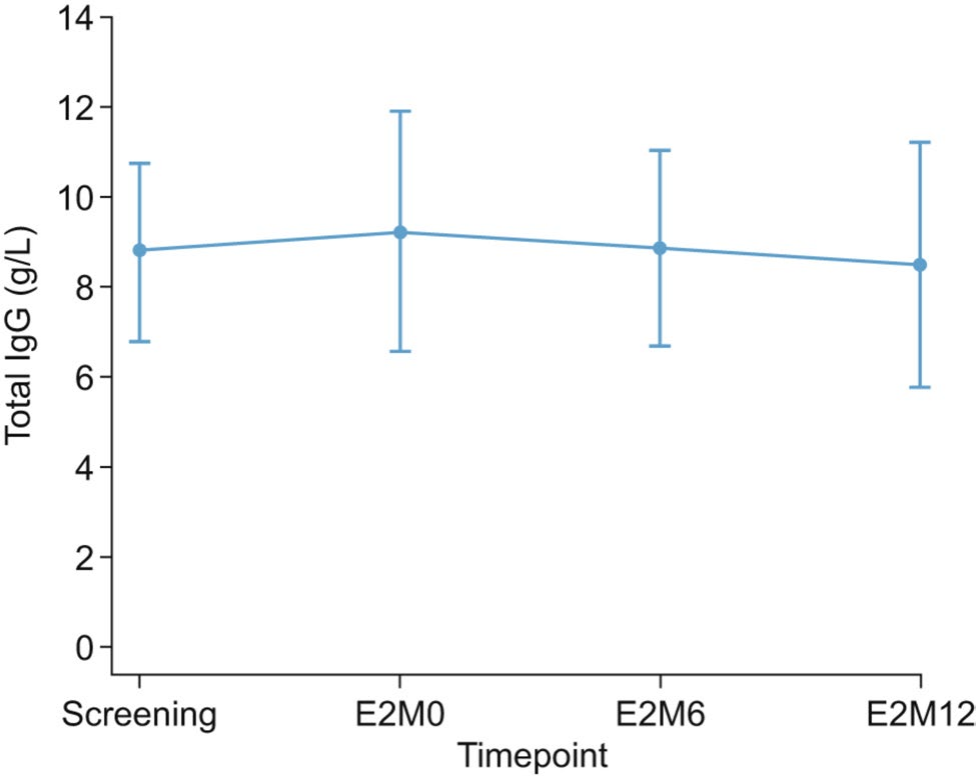
Mean (SD) concentration–time profile of total serum IgG trough levels over time. E2M0, epoch 2 month 0; E2M6, epoch 2 month 6; E2M12, epoch 2 month 12; IgG, immunoglobulin G; SD, standard deviation

Overall, no substantial differences in serum IgG trough levels occurred between patient groups, with levels remaining stable throughout epoch 2 (Fig. 3). Overall mean (SD) and median (range) serum total IgG levels for all 42 patients enrolled and dosed were 8.8 (2.0) g/L and 8.9 (4.9–13.1) g/L at baseline, and 8.5 (2.7) g/L and 8.7 (1.4–15.9) g/L at 1 year. The slight serum IgG trough level decrease observed at month 12 of epoch 2 could be attributed to the critically low serum IgG trough level (1.4 g/L; change from baseline: − 8.0 g/L) developed by an 11-year-old male patient pretreated with fSCIG 10%, potentially due to newly-diagnosed Crohn’s disease (as reported by the treating physician) at the month 12 visit of epoch 2. This event was reported as an unrelated TEAE, and following management of the AE, the patient’s serum IgG trough level had improved by study completion (6.8 g/L).

### Administration characteristics

In total, 795 infusions were administered (464 in new starters; 331 in pretreated patients), with an overall mean (SD) infusion duration of 89.0 (34.5) minutes and mean (SD) number of infusions per patient of 18.9 (5.3). The mean (SD) number of infusions was 1.3 (0.2), with a median (range) of 1.2 (1.0–1.7) infusions per month. The mean (SD) number of infusion sites per month was 1.7 (0.8), and the median (range) number of sites per month was 1.5 (0.3–3.0). Overall, the mean (SD) infusion volume was 139.7 (89.8) mL/site, the mean (SD) maximum infusion rate was 177.6 (83.0) mL/hour/site, and 57.1% of patients received a maximum infusion rate of up to 300 mL/hour/site. In total, 10 patients (23.8%) (6 [26.1%] new starters and 4 [21.1%] pretreated patients) had infusions that changed in rate; 3 patients (7%) (all new starters) had infusions interrupted, and 1 patient (2%) (new starter) had infusions stopped owing to severe AEs (infusion site pain, emotional distress).

### HRQoL and HCRU

At baseline, most patients responding to the Treatment Preference Questionnaire preferred treatment administration at home (14/15 patients; 93.3%); responses were similar among new starters and pretreated patients. At the end of epoch 2, of the 20 patients who responded, all reported that they would choose to continue receiving fSCIG 10% (see Supplementary results, Additional file 1).

Improvements from baseline in some domains and total scores of the 9-item Treatment Satisfaction Questionnaire for Medication, Pediatric Quality of Life Inventory, and European Quality of Life 5-Dimension questionnaires were also observed at the end of epoch 2. No conclusive findings could be determined with regard to HCRU. Further information on HRQoL and HCRU data is provided in Supplementary results, Additional file 1 and Supplementary Tables 1–4, Additional files 2–5.

## Discussion

This European post-authorization safety study demonstrated favorable long-term fSCIG 10% safety and tolerability profiles in pediatric patients with PIDs, consistent with previous studies in this population [10, 12]. No treatment-related SAEs or deaths were reported. AEs and adverse reactions (regardless of severity and causality) were observed at relatively lower proportions among fSCIG 10% pretreated patients than in new starters, suggesting that AEs decreased with increasing exposure to fSCIG 10%. The majority of treatment-related TEAEs were mild, with only two severe treatment-related TEAEs (infusion site pain and emotional distress) reported in one new starter with several ongoing medical conditions.

No patients developed binding anti-rHuPH20 antibodies with a titer of ≥ 1:160, and therefore no patients were assessed for neutralizing anti-rHuPH20 antibodies. This is similar to observations from the pivotal and extension trials of fSCIG 10%, in which no neutralizing anti-rHuPH20 antibodies were noted, and three patients developed transient binding anti-rHuPH20 antibodies with titers comparable to healthy individuals at follow-up visits despite continued rHuPH20 exposure [10].

No substantial difference from baseline in total IgG serum trough levels across all timepoints was observed, and protective IgG levels were maintained throughout epoch 2. fSCIG 10% efficacy was further demonstrated by the low rate of infections and ASBIs per patient, consistent with previous studies [10, 12].

IVIG and conventional SCIG have been shown to have comparable, beneficial safety profiles in patients with PID [16–21], though IVIG treatment carries a greater risk of systemic AEs than subcutaneous therapies [15]. fSCIG 10% combines the benefits of IVIG and SCIG therapies, and can be administered at the same dosing interval as IVIG for PIDs (every 3–4 weeks). Similar to conventional SCIG therapies, fSCIG 10% does not require venous access, and is associated with fewer systemic AEs than IVIG, even in patients experiencing severe systemic effects with intravenous administration [12, 22, 23]. Of the treatment-related AEs observed in the current study, the majority of events were local, and the most commonly reported were infusion site pain, pruritus, and erythema. This is similar to AEs reported in studies evaluating conventional SCIG therapies, which predominantly show mild and self-limiting local reactions at the site of infusion [16–18], though direct comparisons cannot be made in the absence of a head-to-head trial. In contrast, the most frequently observed AEs with IVIG treatment in patients with PIDs have been systemic events such as headache, fatigue, and nausea [16, 19–21, 24]. The opportunity for home administration with fSCIG 10% is also popular with patients, and may reduce overall treatment burden, which is supported by a retrospective chart analysis of pediatric fSCIG 10% home usage [25]. Furthermore, home administration offers reduced exposure to nosocomial infections, versus IVIG administration in a hospital setting [26]. Patients also expressed a strong preference for fSCIG 10%, with most providing positive responses to the majority of questions in the Treatment Preference Questionnaire. Finally, we observed a low rate of hospitalization and length of hospital stay, illustrating potentially wider socioeconomic benefits of fSCIG 10% for both patients and healthcare systems.

Study strengths include patient participation from multiple centers across Europe, the lengthy follow-up period (> 3 years), a substantial duration of fSCIG 10% exposure with a total of 795 infusions administered, and the high study completion rate. As with any safety study, erroneous Medical Dictionary for Regulatory Activities (MedDRA) coding may have occurred; for example, some infections may have been coded without fully capturing associated clinical circumstances. In addition, the relatively small and predominantly White patient sample size of the current study might limit the generalizability of these findings in more diverse populations. However, the pharmacokinetic and pharmacodynamic characteristics of immunoglobulins are considered similar in patients of different races and ethnic backgrounds, and use of fSCIG 10% as IgRT is approved in many countries [7, 8]. 

## Conclusion

In conclusion, we have demonstrated that fSCIG 10% combines the benefits of IVIG and conventional SCIG (including less frequent dosing, more stable trough IgG levels, low incidence of systemic AEs, and the potential for more flexible and personalized treatment regimens) in children with PIDs.

## Electronic supplementary material

Below is the link to the electronic supplementary material.

Supplementary Material 1

Supplementary Material 2

Supplementary Material 3

Supplementary Material 4

Supplementary Material 5

## Data Availability

The data sets, including the redacted study protocol, redacted statistical analysis plan, and individual participant data supporting the results reported in this article, will be made available within 3 months from initial request, to researchers who provide a methodologically sound proposal. The data will be provided after its de-identification, in compliance with applicable privacy laws, data protection, and requirements for consent and anonymization.

## Abbreviations

Ab: Antibody
AE: Adverse event
AR: Autosomal recessive
ASBI: Acute serious bacterial infection
BMI: Body mass index
FAS: Full analysis set
fSCIG: Facilitated subcutaneous immunoglobulin
HCRU: Healthcare resource utilization
HRQoL: Health-related quality of life
IgG: Immunoglobulin G
IgRT: Immunoglobulin replacement therapy
IVIG: Intravenous immunoglobulin
MedDRA: Medical Dictionary for Regulatory Activities
NEMO: Nuclear factor kappa B essential modulator
PI3K: Phosphoinositide 3-kinase
PID: Primary immunodeficiency disease
rHuPH20: Recombinant human hyaluronidase
SAE: Serious adverse event
SAS: Safety analysis set
SCIG: Subcutaneous immunoglobulin
SD: Standard deviation
TEAE: Treatment-emergent adverse event
XLA: X-linked agammaglobulinemia
