# Supplementary material for: fSCIG 10% in pediatric primary immunodeficiency diseases: a European post-authorization safety study
Source: Allergy Asthma Clin Immunol. 2024 Sep 17;20:47. doi: 10.1186/s13223-024-00904-9 (PMC11406826; doi:10.1186/s13223-024-00904-9)
Supplement: Supplementary file 1 — Supplementary Material 1 [file 13223_2024_904_MOESM1_ESM.docx]

# Supplementary methods

## Exclusion criteria

- Known history of, or positive at screening for, one or more of the following: hepatitis B surface antigen, polymerase chain reaction (PCR) for hepatitis C virus, PCR for human immunodeficiency virus type 1/2.
- Abnormal laboratory values at screening, meeting any one of the following criteria (abnormal tests could be repeated once to determine if they were persistent):

1. Persistent alanine aminotransferase and aspartate aminotransferase > 2.5 times the upper limit of normal for the testing laboratory.

b. Persistent severe neutropenia (absolute neutrophil count ≤ 500/mm^3^).

- Anemia that would have precluded phlebotomy for laboratory studies, according to standard practice at the site.
- An ongoing history of hypersensitivity or persistent reactions (urticaria, breathing difficulty, severe hypotension, or anaphylaxis) following intravenous immunoglobulin, subcutaneous immunoglobulin, and/or immune serum globulin infusions.
- Severe immunoglobulin A (IgA) deficiency (< 7.0 mg/dL) with known anti-IgA antibodies and a history of hypersensitivity.
- A known allergy to hyaluronidase.
- An active infection and receiving antibiotic therapy for the treatment of infection at the time of screening.
- A bleeding disorder, or a platelet count < 20,000/μL, or a patient who, in the opinion of the investigator, would have been at significant risk of increased bleeding or bruising as a result of subcutaneous therapy.
- Severe dermatitis that would have precluded adequate sites for safe product administration in the opinion of the investigator.
- Participated in another clinical study involving an investigational product (IP) or investigational device in the 30 days before enrollment or was scheduled to participate in another clinical study involving an IP or investigational device during the course of this study.
- A family member or an employee of the investigator.
- If female, patient was pregnant or lactating at the time of enrollment.

## Secondary and tertiary outcomes

- Efficacy: serum trough levels of immunoglobulin G (IgG) in epochs 1 and 2.
- Safety and tolerability: number and rate per infusion (excluding infections) of all adverse events (AEs) and serious adverse events (SAEs), adverse reactions (ARs) (i.e. events deemed “possibly related” or “probably related” to hyaluronidase-facilitated subcutaneous immunoglobulin [fSCIG] 10%), and all related/temporally associated AEs and SAEs.
- Immunogenicity: proportion of patients developing a positive titer ≥ 1:160 of binding or neutralizing antibodies to rHuPH20.
- Treatment administration characteristics: number of infusions per month, number of infusion sites, infusion rates and volumes, and proportion of interrupted infusions.
- Health-related quality of life (HRQoL), assessed using a treatment preference questionnaire, a treatment satisfaction questionnaire (TSQM-9) and HRQoL questionnaires (Pediatric Quality of Life Inventory [Peds-QL] and European Quality of Life 5 Dimension [EQ-5D]).
- Number of all infections and acute serious bacterial infections (ASBIs).
- Healthcare resource utilization (HCRU), including ability to go to school or work, to perform normal daily activities, days on antibiotics, hospitalizations, and acute physician visits.

## Statistical analysis

- All secondary safety outcomes were analyzed using descriptive methods.
- Efficacy outcome measures (serum trough levels of total IgG, IgG subclass, and specific antibodies) were analyzed using descriptive statistics, including geometric mean.
- A point estimate and 95% confidence intervals determined by the Wilson score method were used to analyze the incidence of all infections and ASBIs. In addition, the incidence of all infection endpoints was calculated as a rate per infusion and a rate per patient-year.
- Nonparametric descriptive statistics (median, quartiles, and range) were used to analyze administration variables.
- For other analyses, including HRQoL and HCRU, descriptive statistics were calculated and reported.

# Supplementary results

## Additional details of patient discontinuing epoch 2 and entering epoch 3

- During fSCIG 10% treatment in epoch 2, one patient, an 11-year-old white male, presented with severe infusion site pain and severe emotional distress.
  - The patient had a medical history that included self-harming and several ongoing medical conditions including developmental delay, anxiety, and attention deficit hyperactivity disorder.
- fSCIG 10% treatment was withdrawn and the patient entered epoch 3. The infusion site pain resolved on the same day of onset while the emotional distress resolved within 3 days.

## Health-related quality of life and healthcare resource utilization

### HRQoL

- At the end of epoch 2, data on treatment preference were available from 20 patients in the overall study population. All of these patients indicated that they would choose to continue fSCIG 10% treatment, with a similar response between fSCIG 10% new starters and pretreated patients.
- Across the three domains of the TSQM-9 evaluated, an improvement in the convenience domain was observed at the end of epoch 2 compared with baseline in the overall study population (**Table S1**); this improvement was numerically greater among fSCIG 10% new starters whereas scores remained stable from baseline to end of epoch 2 among the pretreated population. However, data for TSQM-9 should be interpreted with caution owing to the overall small sample size at the end of epoch 2 (*n =*23 for the effectiveness evaluation and *n =*24 each for the convenience and global satisfaction evaluation).
- Peds-QL scores at baseline and at the end of epoch 2 according to different age group categories are summarized in **Table S2**.
- EQ-5D index and EQ-Visual Analogue Scale scores at baseline and at the end of epoch 2 are summarized in **Table S3**.
- Results for both assessments should be interpreted with caution owing to the small sample size and volume of missing data.

### HCRU

- In total, 39 patients were analyzed for HCRU: 22 fSCIG 10% new starters and 17 fSCIG 10% pretreated patients (**Table S4**).
- Overall, patients were unable to attend school/work or perform normal daily activities owing to infection or other illness at a rate of 9.4 days per patient and 7.5 days per patient-year. These rates were lower for new starters (8.0 days per patient and 6.3 days per patient-year) compared with the pretreated population (11.0 days per patient and 9.1 days per patient-year).
- Approximately half of the overall population received antibiotic therapy during fSCIG 10% treatment (*n =*23 [54.8%]; mean [standard deviation (SD)] duration: 146.1 [202.6] days per patient). Higher antibiotic usage was observed among pretreated patients (*n =*13 [68.4%]; mean [SD] duration: 154.2 [210.6] days per patient) than in new starters (*n =*10 [43.5%]; mean [SD] duration:135.5 (202.5) days per patient).
- Hospitalizations occurred for 7 patients overall (2 new starters and 5 pretreated patients), with a lower mean (SD) number of hospitalizations and number of days hospitalized among new starters (1.0 [0.0]; 3.5 [0.7] days) than in pretreated individuals (1.4 [0.6]; 5.4 [2.4]).
