# Supplementary material for: fSCIG 10% in pediatric primary immunodeficiency diseases: a European post-authorization safety study
Source: Allergy Asthma Clin Immunol. 2024 Sep 17;20:47. doi: 10.1186/s13223-024-00904-9 (PMC11406826; doi:10.1186/s13223-024-00904-9)
Supplement: Supplementary file 2 — Supplementary Material 2 [file 13223_2024_904_MOESM2_ESM.docx]

**Supplementary Table 1** TSQM-9 domain scores at baseline and end of epoch 2

| **TSQM-9 domain** | **fSCIG 10% new starters**  **(*n =*23)** | **fSCIG 10% pretreated**  **(*n =*19)** | **Total**  **(*N =*42)** |
| --- | --- | --- | --- |
| Effectiveness, mean (SD)  Baseline  End of epoch 2  Change from baseline | *n = 5/n = 13*  76.7 (27.3)  70.9 (26.9)  13.0 (32.6) | *n = 8/n = 10*  85.4 (10.7)  82.8 (14.2)  –0.9 (10.8) | *n = 13/n = 23*  82.1 (18.3)  76.1 (22.7)  3.7 (19.6) |
| Convenience, mean (SD)  Baseline  End of epoch 2  Change from baseline | *n = 5/n = 13*  55.6 (35.4)  73.5 (15.4)  14.8 (27.4) | *n = 8/n = 11*  75.7 (7.8)  74.2 (13.2)  4.6 (15.9) | *n = 13/n = 24*  68.0 (23.6)  73.8 (14.1)  8.0 (19.3) |
| Global satisfaction, mean (SD)  Baseline  End of epoch 2  Change from baseline | *n = 5/n = 13*  77.1 (13.7)  78.6 (16.2)  11.9 (10.9) | *n = 8/n = 11*  88.4 (6.5)  81.8 (17.6)  –2.4 (19.5) | *n = 13/n = 24*  84.1 (11.0)  80.1 (16.6)  2.4 (17.9) |

Numbers of patients at baseline/end of epoch 2 are given. Higher scores indicate higher treatment satisfaction

fSCIG, hyaluronidase-facilitated subcutaneous immunoglobulin; SD, standard deviation; TSQM-9, Treatment Satisfaction Questionnaire for Medication (9-item)
