# Supplementary material for: fSCIG 10% in pediatric primary immunodeficiency diseases: a European post-authorization safety study
Source: Allergy Asthma Clin Immunol. 2024 Sep 17;20:47. doi: 10.1186/s13223-024-00904-9 (PMC11406826; doi:10.1186/s13223-024-00904-9)
Supplement: Supplementary file 3 — Supplementary Material 3 [file 13223_2024_904_MOESM3_ESM.docx]

**Supplementary Table 2** Peds-QL total score at baseline and end of epoch 2 by age group

|  | **fSCIG 10% new starters**  **(*n =*23)** | **fSCIG 10% pretreated**  **(*n =*19)** | **Total**  **(*N =*42)** |
| --- | --- | --- | --- |
| Age group: 2–4 years, mean (SD)  Baseline  End of epoch 2  Change from baseline | *n = 0/n = 1*  –  67.5 (–)  – | *n = 2/n = 2*  96.4 (0.0)  86.9 (16.8)  2.4 (–) | *n = 2/n = 3*  96.4 (0.0)  80.4 (16.3)  2.4 (–) |
| Age group: 5–7 years, mean (SD)  Baseline  End of epoch 2  Change from baseline | *n = 0/n = 2*  –  63.6 (13.1)  – | *n = 0/n = 1*  –  63.0 (–)  –33.4 (–) | *n = 0/n = 3*  –  63.4 (9.2)  –33.4 (–) |
| Age group: 8–12 years, mean (SD)  Baseline  End of epoch 2  Change from baseline | *n = 5/n = 4*  60.8 (22.3)  71.5 (20.5)  –3.3 (3.1) | *n = 2/n = 2*  79.4 (27.7)  58.0 (16.0)  –52.2 (–) | *n = 7/n = 6*  66.1 (23.2)  67.0 (18.7)  –19.6 (28.3) |
| Age group: 13–<18 years, mean (SD)  Baseline  End of epoch 2  Change from baseline | *n = 3/n = 7*  85.5 (6.4)  86.4 (8.8)  –6.4 (6.5) | *n = 6/n = 5*  76.2 (15.6)  73.3 (11.4)  3.2 (14.5) | *n = 9/n = 12*  79.3 (13.6)  80.9 (11.6)  –0.4 (12.5) |

Numbers of patients at baseline/end of epoch 2 are given. Higher scores indicate better quality of life

fSCIG, hyaluronidase-facilitated subcutaneous immunoglobulin; Peds-QL, Pediatric Quality of Life Inventory; SD, standard deviation
