# Supplementary material for: fSCIG 10% in pediatric primary immunodeficiency diseases: a European post-authorization safety study
Source: Allergy Asthma Clin Immunol. 2024 Sep 17;20:47. doi: 10.1186/s13223-024-00904-9 (PMC11406826; doi:10.1186/s13223-024-00904-9)
Supplement: Supplementary file 4 — Supplementary Material 4 [file 13223_2024_904_MOESM4_ESM.docx]

**Supplementary Table 3** EQ-5D index and EQ-VAS scores at baseline and end of epoch 2

|  | **fSCIG 10% new starters**  **(*n =*23)** | **fSCIG 10% pretreated**  **(*n =*19)** | **Total**  **(*N =*42)** |
| --- | --- | --- | --- |
| EQ-5D index, mean (SD)  Baseline  End of epoch 2  Change from baseline | *n = 7/n = 12*  0.88 (0.25)  0.94 (0.10)  –0.09 (0.08) | *n = 9/n = 9*  0.88 (0.10)  0.90 (0.12)  0.03 (0.15) | *n = 16/n = 21*  0.88 (0.18)  0.92 (0.11)  –0.02 (0.14) |
| EQ-VAS score, mean (SD)  Baseline  End of epoch 2  Change from baseline | *n = 7/n = 12*  92.6 (2.8)  87.6 (11.1)  –8.6 (11.1) | *n = 9/n = 9*  84.0 (12.0)  79.8 (10.4)  –4.4 (15.4) | *n = 16/n = 21*  87.8 (10.0)  84.2 (11.3)  –6.2 (13.4) |

Numbers of patients at baseline/end of epoch 2 are given. Higher scores indicate better quality of life

EQ-5D, European Quality of Life 5 Dimension; EQ-VAS, European Quality Visual Analogue Scale; fSCIG, hyaluronidase-facilitated subcutaneous immunoglobulin; SD, standard deviation
