# Supplementary material for: fSCIG 10% in pediatric primary immunodeficiency diseases: a European post-authorization safety study
Source: Allergy Asthma Clin Immunol. 2024 Sep 17;20:47. doi: 10.1186/s13223-024-00904-9 (PMC11406826; doi:10.1186/s13223-024-00904-9)
Supplement: Supplementary file 5 — Supplementary Material 5 [file 13223_2024_904_MOESM5_ESM.docx]

**Supplementary Table 4** Healthcare resource utilization

| **HCRU parameter** | **fSCIG 10% new starters**  **(*n =*23)** | **fSCIG 10% pretreated**  **(*n =*19)** | **Total**  **(*N =*42)** |
| --- | --- | --- | --- |
| Patients with available data, *n* | 22 | 17 | 39 |
| Days unable to attend school/work or perform normal daily activities owing to infection/other illness, mean (SD) | 8.4 (14.3) | 12.3 (13.5) | 10.1 (13.9) |
| Patients with any antibiotic use, *n* (%) | 10 (43.5) | 13 (68.4) | 23 (54.8) |
| Days on antibiotics, mean (SD) | 135.5 (202.5) | 154.2 (210.6) | 146.1 (202.6) |
| Outpatient visits, mean (SD) | 1.5 (2.0) | 3.7 (6.1) | 2.5 (4.4) |
| Acute physician visits, mean (SD) | 0.8 (1.4) | 1.3 (1.4) | 1.0 (1.4) |
| Hospitalizations, mean (SD) | 1.0 (0.0) | 1.4 (0.6) | 1.3 (0.5) |
| Days hospitalized, mean (SD) | 3.5 (0.7) | 5.4 (2.4) | 4.9 (2.2) |

fSCIG, hyaluronidase-facilitated subcutaneous immunoglobulin; HCRU, healthcare resource utilization; SD, standard deviation
